# Rationale for the selection of dual primary endpoints in prevention studies of cognitively unimpaired individuals at genetic risk for developing symptoms of Alzheimer’s disease

**DOI:** 10.1186/s13195-023-01183-z

**Published:** 2023-03-06

**Authors:** Angelika Caputo, Amy Racine, Ines Paule, Pierre N. Tariot, Jessica B. Langbaum, Neva Coello, Marie-Emmanuelle Riviere, J. Michael Ryan, Cristina Lopez Lopez, Ana Graf, for the National Alzheimer’s Coordinating Center, for the Alzheimer’s Disease Neuroimaging Initiative

**Affiliations:** 1 Novartis Pharma AG, PostfachCH-4002, Basel, Switzerland; 2 Banner Alzheimer’s Institute, Phoenix, AZ USA; 3 Aliada Therapeutics, Boston, MA USA

**Keywords:** Alzheimer’s disease, Preclinical phase, Cognitively unimpaired, *APOE* genotype, Dual endpoints, Time to event, APCC, Model-informed drug development, Clinical trial simulation

## Abstract

**Background:**

There is a critical need for novel primary endpoints designed to detect early and subtle changes in cognition in clinical trials targeting the asymptomatic (preclinical) phase of Alzheimer’s disease (AD). The Alzheimer’s Prevention Initiative (API) Generation Program, conducted in cognitively unimpaired individuals at risk of developing AD (e.g., enriched by the *apolipoprotein E (APOE)* genotype), used a novel dual primary endpoints approach, whereby demonstration of treatment effect in one of the two endpoints is sufficient for trial success. The two primary endpoints were (1) time to event (TTE)—with an event defined as a diagnosis of mild cognitive impairment (MCI) due to AD and/or dementia due to AD—and (2) change from baseline to month 60 in the API Preclinical Composite Cognitive (APCC) test score.

**Methods:**

Historical observational data from three sources were used to fit models to describe the TTE and the longitudinal APCC decline, both in people who do and do not progress to MCI or dementia due to AD. Clinical endpoints were simulated based on the TTE and APCC models to assess the performance of the dual endpoints versus each of the two single endpoints, with the selected treatment effect ranging from a hazard ratio (HR) of 0.60 (40% risk reduction) to 1 (no effect).

**Results:**

A Weibull model was selected for TTE, and power and linear models were selected to describe the APCC score for progressors and non-progressors, respectively. Derived effect sizes in terms of reduction of the APCC change from baseline to year 5 were low (0.186 for HR = 0.67). The power for the APCC alone was consistently lower compared to the power of TTE alone (58% [APCC] vs 84% [TTE] for HR = 0.67). Also, the overall power was higher for the 80%/20% distribution (82%) of the family-wise type 1 error rate (alpha) between TTE and APCC compared to 20%/80% (74%).

**Conclusions:**

Dual endpoints including TTE and a measure of cognitive decline perform better than the cognitive decline measure as a single primary endpoint in a cognitively unimpaired population at risk of AD (based on the *APOE* genotype). Clinical trials in this population, however, need to be large, include older age, and have a long follow-up period of at least 5 years to be able to detect treatment effects.

**Supplementary Information:**

The online version contains supplementary material available at 10.1186/s13195-023-01183-z.

## Background

Clinical trials in Alzheimer’s disease (AD) have a high failure rate. An analysis of the drug pipeline over two decades showed an attrition rate of nearly 100% [1, 2]. Possible explanations for these failures include insufficient evidence to initiate pivotal trials and pivotal trial design shortcomings such as selection of suboptimal drug dosage levels, wrong treatment targets, inappropriate study population, and inadequate understanding of the biology of AD [2, 3].

Failure to detect an effect may also result from assessing the drug in patients who are in later stages of the disease process, that is, after symptoms have appeared [4]. The design of clinical trials in cognitively unimpaired individuals has its own particular challenges, including the lack of sensitive tools or scales to detect early changes, the absence of surrogate biomarkers, combined with the long duration required to detect progression to initial clinical symptoms of AD (i.e., cognitive impairment). Novel primary endpoints designed to detect early and subtle changes in cognition thus constitute a critical need for any clinical trial targeting the preclinical phase of the disease [5]. Supporting the design of such endpoints, Anderson et al. suggested that clinical trial simulations could be a powerful way to provide insight into the likelihood of a trial succeeding [6, 7].

The Alzheimer’s Prevention Initiative (API) Generation Program comprised two pivotal phase 2/3 studies, Generation Study 1 (NCT02565511) and Generation Study 2 (NCT03131453), specifically designed to assess the efficacy and safety of investigational drugs in a cognitively unimpaired population identified as being at an increased risk of developing AD symptoms. Such risk was determined based on age, apolipoprotein E (*APOE*) genotype, and brain amyloid level (i.e., the presence of two *APOE* ε4 [*APOE4*] alleles in Generation Study 1 and of at least one *APOE4* allele in Generation Study 2 together with elevated brain amyloid level in those with one allele) [8].

In an effort to devise endpoints suitable for prevention studies, the API Generation Program used a novel approach: dual primary endpoints based on (1) time to event (TTE)—with an event defined as a diagnosis of mild cognitive impairment (MCI) due to AD and/or dementia due to AD—and (2) change from baseline to month 60 in the API preclinical composite cognitive (APCC) test score—with the APCC designed to detect and track longitudinal cognitive decline in individuals with preclinical AD [9]. In such an approach, the demonstration of a treatment effect on one of the two distinct primary endpoints is sufficient for trial success. As such, the dual endpoints approach afforded the potential to capture both early and subtle cognitive changes (with a continuous measure: APCC) and discrete clinically relevant events (with TTE).

The selection of the dual endpoints was based on the exploration of historical data followed by the tailored clinical trial simulation of Generation Study 1. This two-step process allowed the investigation and power optimization of various design options with regard to endpoints, sample size, duration, and population characteristics, among others. Here, we present (1) the rationale for the dual endpoints approach, (2) its validation by longitudinal model-based analysis of historical cohort data and clinical trial simulation, and (3) results from the simulated clinical trials and how these results informed the clinical study design.

## Methods

### Historical cohort data

The data used in the preparation of this article were obtained from (1) three cohort studies of aging and dementia from the Rush Alzheimer’s Disease Center (Religious Orders Study [ROS], Memory and Aging Project [MAP], and the Minority Aging Research Study [MARS] cohorts described previously) [10, 11] (https://www.radc.rush.edu), referred to hereafter as the ROS/MAP/MARS cohorts; (2) a clinical case series from the Alzheimer’s Disease Research Centers of the National Alzheimer’s Coordinating Center (NACC) [12] (https://www.alz.washington.edu); and (3) the Alzheimer’s Disease Neuroimaging Initiative (ADNI) database [13] (https://adni.loni.usc.edu).

The ADNI was launched in 2003 as a public-private partnership, led by principal investigator Michael W. Weiner, MD. The primary goal of ADNI has been to test whether serial magnetic resonance imaging (MRI), positron emission tomography (PET), other biological markers, and clinical and neuropsychological assessment can be combined to measure the progression of MCI and early AD.

Baseline characteristics, including genetic information, diagnostic classification, and results from neurocognitive testing, were considered in the exploration of historical data and included in the modeling. Biomarker data, including amyloid status, were only available from ADNI and were therefore not included. The models were fitted to data from the subset of participants who were cognitively unimpaired at the first visit (study entry). Participants with data from at least two visits and who did not develop any type of dementia other than due to AD were included in the longitudinal analyses. Data from the ADNI participants (with non-missing *APOE* genotype) were included in the TTE exploration, but not in other explorations or in the modeling due to the small number of *APOE4* homozygotes in ADNI, combined with the relatively short follow-up time, and the lack of data in some cognitive measures to implement a good proxy to the APCC.

### Dual primary endpoints approach

The success of a trial using a dual endpoints approach is defined by a positive result, i.e., demonstration of treatment effects, in at least one of two distinct primary endpoints. The approach that was selected for use in the Generation Program includes adequate adjustment of the type 1 error rate to account for testing multiple hypotheses and was accepted by both the United States Food and Drug Administration (FDA) and the European Medicines Agency (EMA) [8]. A dual endpoints approach contrasts with a co-primary endpoints approach where the treatment effect has to be demonstrated in both primary endpoints. It also differentiates from so-called combined assessment approaches where typically a TTE and a clinical outcome score are combined into a single ranked outcome variable [14]. In our study, the dual primary endpoints were (1) TTE and (2) change from baseline to month 60 in the APCC test score [9]. In the TTE component, an event was defined as an adjudicated diagnosis of MCI due to AD and/or dementia due to AD, with the diagnosis given at two consecutive visits to ensure it is stable. Strictly speaking, the TTE endpoint is a composite endpoint in the context of the FDA Draft Guidance to industry (2017) [15] insofar as it is defined as the time to the occurrence of any of the two components, i.e., diagnosis of MCI or dementia.

The APCC was developed as a multi-component endpoint [15] to track early cognitive changes in preclinical AD, covering different cognitive domains based on measures collected across the ROS/MAP/MARS cohorts [9]. A proxy was used for NACC as not all the tests comprising the APCC were available (Table 1). The selection of cognitive measures collected in ADNI was deemed insufficient to define a proxy for the APCC in this cohort.

**Table 1:** Cognitive assessments in ROS/MAP/MARS and NACC used in the different composite scores

Scale	ROS/MAP/MARS				NACC			
	Available	APCC	PACC proxy	RBANS proxy	Available	APCC proxy	PACC proxy	RBANS proxy
Boston Naming Test	X (15 items)			X	X (30 items)	X		X
Category fluency – Animals	X			X	X	X		X
Category fluency – Fruits/Vegetables	X			X	X	X		X
CERAD Word List Recall (immediate)	X			X				
CERAD Word List Memory (delayed recall)	X	X	X	X				
CERAD Word List Recognition	X			X				
East Boston Naming Test, Immediate Recall (memory I)	X							
East Boston Naming Test, Delayed Recall (memory II)	X							
Logical Memory Ia (immediate)	X			X	X			X
Logical Memory IIa (delayed)	X	X	X	X	X	X	X	X
Digit Span – Forward	X			X	X			X
MMSE – Total	X		X		X		X	
MMSE – Orientation to time	X	X			X	X		X
MMSE – Orientation to place	X	X			X	X		X
Ravens Progressive Matrices Subset (9 items)	X	X		X				
Judgment of Line Orientation	X	X		X				
Symbol Digit Modalities Test	X	X	X	X				
WAIS Digit Symbol Substitution					X	X	X	X

*APCC* Alzheimer’s Prevention Initiative Preclinical Composite Cognitive, *CERAD* Consortium to Establish a Registry for Alzheimer’s Disease, *MMSE* Mini-Mental State Examination, *NACC* National Alzheimer’s Coordinating Center, *PACC* Preclinical Alzheimer’s Cognitive Composite, *RBANS* Repeatable Battery for the Assessment of Neuropsychological Status, *ROS/MAP/MARS* Religious Orders Study/Memory and Aging Project/Minority Aging Research Study, *WAIS* Wechsler Adult Intelligence Scale

Two additional multi-component endpoints proposed in the literature were also explored. These were the Preclinical Alzheimer’s Cognitive Composite (PACC [16];) and the Repeatable Battery for the Assessment of Neuropsychological Status (RBANS [17];) (see Table 1 for the definition of their proxies for ROS/MAP/MARS and NACC). The multi-component endpoints—APCC, PACC, and RBANS—are referred to as composite cognitive endpoints (or composites in short) hereafter.

To allow for comparisons across the three composites, standardized *z*-scores were derived and used for visual inspection, complemented by a mean-to-standard deviation ratio (MSDR) approach.

### Data exploration

Graphical exploration of historical data was used to develop the modeling and simulation strategies. Three subpopulations of those who were cognitively unimpaired at study entry were explored: progressors to dementia, defined as those who were diagnosed with dementia due to AD at any time during the observation period; progressors to MCI, defined as those who were diagnosed with MCI (but not dementia) during the observation period; and non-progressors to MCI or dementia, defined as those who were not diagnosed with MCI or dementia due to AD during the observation period. Individuals who had a diagnosis of MCI followed by a later diagnostic classification of being cognitively unimpaired at any time after the initial diagnosis without a later diagnosis of dementia were excluded from data explorations, except for the initial event risk estimation to support sample size estimation of Generation Study 1.

#### Exploratory analyses of the TTE

Kaplan-Meier (KM) analyses were performed to estimate the event risk in *APOE4* carriers based on longitudinal data from NACC, ADNI, and ROS/MAP/MARS. An event was defined as the first diagnosis of MCI or dementia due to AD (whichever came first). For participants without a diagnosis of MCI or dementia due to AD, the TTE refers to the time until their last assessment in the study (censoring time).

#### Exploratory analyses of the APCC score

The APCC explorative analyses were based mainly on longitudinal data from the ROS/MAP/MARS cohorts (where all components of the APCC were available; hence, no proxy was needed) and supported by explorations of NACC and of other composites as described in Table 1.

Individual longitudinal APCC data in the three subpopulations defined earlier were visualized by line plots using a time scale anchored to the time of the first diagnosis of dementia due to AD for the progressors to dementia subpopulation. In these plots, data from individuals without a dementia diagnosis (progressors to MCI and non-progressors) were plotted anchored to the median age of progressors to dementia at the time of the first dementia diagnosis. Locally weighted least squares regression curves (otherwise known as locally estimated scatterplot smoothing [LOESS]) were superimposed on the data to visualize and compare the trends among the different subpopulations and data sources.

### Modeling strategy

Models were selected for the two endpoints of Generation Study 1 to allow for the simulation of a clinical trial of 5 to 8 years in duration. The modeling strategy was supported by results from the data exploration. In this modeling phase, two subpopulations were defined: progressors who were cognitively unimpaired at study entry and diagnosed with MCI or dementia due to AD, and non/late progressors who were not diagnosed with MCI or dementia due to AD during the observation period or who were diagnosed later than 8 years after study entry (in this case, only data from the first 8 years were used in model estimation). A two-step modeling approach was used: (1) modeling of TTE and (2) modeling of APCC trajectories in subpopulations defined by their categorized TTE. This approach aimed to capture the correlation between the dual endpoints measured on the same patient, which will be important for simulations of future clinical trials.

Akaike’s Information Criterion (AIC) was used to compare model candidates, whereby the model with the lower AIC score is the model providing the better fit. The adequacy of the selected model was assessed via a visual predictive check (VPC) [18]. For the APCC model, the normalized prediction distribution errors (NPDE) were also used to assess the quality of the model, where each observation is compared to its predictive distribution under the assumed model.

The software used was R package version 3.4.3, SAS version 9.4 for the TTE model, and NONMEM version 7.3.0 for the APCC model (with the first-order conditional estimation with interaction [FOCEI] algorithm).

#### Selection of the TTE model structure

The TTE model was fitted to longitudinal data from NACC and ROS/MAP/MARS, without any restriction on age or genotype. For future predictions and simulations of TTE, we considered the following parametric survival functions: Weibull, piece-wise exponential, exponential, and Gompertz. Two potential outcomes were considered for each participant: (1) diagnosis of MCI or dementia due to AD and (2) diagnosis of dementia due to AD. The same model structure, including the type of event as a factor in the model, was used to allow for estimations of the hazard and impact of covariates on both event types (see Additional file 1 for more details).

#### Selection of the APCC model structures

Data from the ROS/MAP/MARS cohorts were used to fit the APCC models, without any restriction on age or *APOE* genotype. The model structures chosen to describe the change in APCC over time in the two subpopulations of progressors and non/late progressors were mixed effects models (also named hierarchical or population models) under the simplistic assumption of no correlations among individual random effects. The two longitudinal models used different time reference points: in the progressors model, “time = 0” is 12 years before the first event (MCI or dementia due to AD), and in the non/late progressors model, “time = 0” is the start of the observation period. Both models used logit transformation to constrain the endpoint values between 0 and 100.

#### Selection of covariates

Clinically relevant variables, including sex, baseline age, years of education, baseline APCC score, and *APOE* genotype, were considered in the exploratory phase as candidate covariates. Among these, the variables with a potential association with the outcome were tested for inclusion in both the APCC and TTE models using backward elimination based on the AIC criterion. Brain amyloid elevation status could not be used as a covariate as this information was not available in the ROS/MAP/MARS cohorts. Participants with unknown *APOE* genotypes (not genotyped or missing) were considered to be non-carriers. Participants with missing values for other important baseline variables were not included in the modeling step.

### Simulation strategy

The simulation was performed according to the following stepwise procedure: (1) The multivariate distribution of characteristics of a population of virtual subjects was informed by available clinical trial data (age, sex, race, and body weight) and by NACC and ROS/MAP/MARS (genotype, educational level, and baseline APCC as a measure of cognitive status depending on age and educational level). Some of the information included in the simulation was not needed to inform the selection of the endpoint (e.g., sex, race, and body weight) but was used for other purposes not relevant to the work described here. (2) For the simulated subjects, the time to the first event (MCI or dementia due to AD) was simulated according to the TTE model. The hazard ratio (HR) was selected to be between 0.60 (40% risk reduction on active treatment) and 1.0 (no treatment effect, placebo), with a HR < 1 reflecting different hypothesized beneficial treatment effect sizes of the active arm versus placebo (HRs of 0.60, 0.65, 0.67, 0.70, and 0.75). The outcome of the TTE simulation was used to classify subjects as either non/late progressors (no event or TTE > 8 years) or progressors (TTE ≤ 8 years) and to identify the time point of progression. (3) Cognitive decline was then simulated according to the APCC models for the simulated non/late progressors or progressors (every 6 months over 8 years). (4) Clinical trials were generated by sampling from the pool of simulated subjects according to specified (scenario-specific) assumptions on baseline characteristics (such as age distribution) and treatment effect. The simulation platform also allowed for the investigation of various recruitment and drop-out patterns (data not shown).

### Simulation of Generation Study 1

The main goal of the simulations was to assess the performance of the dual endpoints versus each of the two endpoints if they were selected as a single primary endpoint in Generation Study 1. The target population was defined as cognitively unimpaired *APOE4* homozygotes aged 60–75 years. Following the aforementioned steps, a set of about 18,000 virtual subjects for each active arm per assumed HR and about 25,000 virtual subjects for placebo were simulated.

The simulation platform allowed us to experiment with various clinical trial design scenarios (in terms of sample size, age distribution within the target age range, drop-out rates and patterns, etc.) to understand the impact of these parameters on the event rates, on the speed of cognitive decline, and on the power to identify an existing treatment effect on at least one of the two endpoints. Power was calculated using a simple Bonferroni adjustment of the family-wise type 1 error rate of 5% for the following design scenarios: 100% vs 0% (single primary endpoint: TTE), 80% vs 20%, 50% vs 50%, 20% vs 80%, and 0% vs 100% (single primary endpoint: APCC) for TTE vs APCC, respectively. The power using the dual endpoints approach at the projected time of analysis (when the last participant reaches 5 years of follow-up) was estimated based on 1000 simulated clinical trials and appropriate two-sided statistical tests. For a fair comparison of power, these 1000 clinical trials were simulated with a fixed total sample size of 650 and a randomization ratio of 3:2 for the active versus control arms under the simple assumptions of no drop-outs and a fixed follow-up duration of 5 years for each participant.

## Results

### Data exploration

#### Baseline characteristics and outcomes

Our investigations were based on data from ADNI, NACC, and ROS/MAP/MARS, including 10,390 initially cognitively unimpaired participants with one baseline and at least one post-baseline visit. The observed characteristics and main outcomes of the included participants are given in Table 2.

**Table 2:** Baseline characteristics and outcomes of initially cognitively unimpaired individuals

	ROS/MAP/MARS, ***N*** = 1682	NACC, ***N*** = 8218	ADNI, ***N*** = 490
Sex, *n* (%)			
Female	1272 (75.6%)	5326 (64.8%)	253 (51.6%)
*APOE4* status, *n* (%)			
Homozygote	29 (1.7%)	147 (1.8%)	11 (2.2%)
Heterozygote	285 (16.9%)	1747 (21.3%)	131 (26.7%)
Non-carrier	1033 (61.4%)	4634 (56.4%)	348 (71.0%)
Unknown/not genotyped	335 (19.9%)	1690 (20.6%)	Not included
Progressed during the observation period, *n* (%)			
To MCI	462 (27.5%)	1307 (15.9%)	80 (16.3%)
To dementia due to AD	290 (17.2%)	569 (6.9%)	25 (5.1%)
To MCI or dementia due to AD	497 (29.6%)	1579 (19.2%)	80 (16.3%)
Progressed within 8 years, *n* (%)			
To MCI	360 (21.4%)	1305 (15.9%)	71 (14.5%)
To dementia due to AD	170 (10.1%)	568 (6.8%)	14 (2.9%)
To MCI or dementia due to AD	389 (23.1%)	1577 (19.2%)	73 (14.9%)
Age at study entry in years			
Mean (SD)	76.2 (7.4)	73.9 (8.2)	74.3 (5.8)
Age category, *n* (%)			
Below 60	Not included	Not included	4 (0.8%)
60 to < 65	56 (3.3%)	1116 (13.6%)	10 (2.0%)
65 to < 70	359 (21.3%)	1593 (19.4%)	91 (18.6%)
70 to < 75	358 (21.3%)	1776 (21.6%)	168 (34.3%)
75 years of age or older	909 (54.0%)	3733 (45.4%)	217 (44.3%)
Years of education			
Median (interquartile range)	16 (13–18)	16 (13–18)	16 (14–18)
Follow-up in years			
Median (interquartile range)	6 (3–10)	4 (2–6)	3.5 (2–5)

*AD* Alzheimer’s Disease, *ADNI* Alzheimer’s Disease Neuroimaging Initiative, *APOE4* apolipoprotein E ε4, *MCI* mild cognitive impairment, *N* total number of cognitively unimpaired individuals who had at least one post-baseline visit and did not have a diagnosis of MCI or dementia due to AD at study entry in the corresponding cohort, *NACC* National Alzheimer’s Coordinating Center, *ROS/MAP/MARS* Religious Orders Study/Memory and Aging Project/Minority Aging Research Study, *SD* standard deviation

#### Exploration of the TTE

We explored data from ADNI, NACC, and ROS/MAP/MARS to estimate the 5-year risk of a first diagnosis of MCI or dementia due to AD for the target population of 60- to 75-year-old *APOE4* homozygotes of Generation Study 1. The estimation was based on data from 5108 genotyped participants aged 60–75 years out of which 1478 (29%) were heterozygotes and 146 (3%) were homozygotes. The estimated event risk (KM estimate) in the pooled ROS/MAP/MARS, NACC, and ADNI populations was higher for *APOE4* homozygotes than for heterozygotes and non-carriers, in agreement with previous observations of the increased risk of (an earlier) onset of symptoms of cognitive impairment in *APOE4* homozygotes [19]: 38% for *APOE4* homozygotes, 23% for heterozygotes, and 16% for non-carriers.

Assuming a treatment effect of a risk reduction of 33% (i.e., HR of 0.67), 218 observed events are needed to reach a power of at least 80% based on the Schoenfeld formula [20]. A total sample size of 650 with a randomization ratio of 3:2 for active versus control treatment was planned for Generation Study 1 to reach 80% power to demonstrate a treatment effect on the TTE endpoint on its own using a two-sided test and a type 1 error rate of 4% when the last randomized participant reaches the 5-year follow-up time assuming a 30% drop-out rate in 5 years.

#### Exploration of the APCC score

**Fig. 1:**
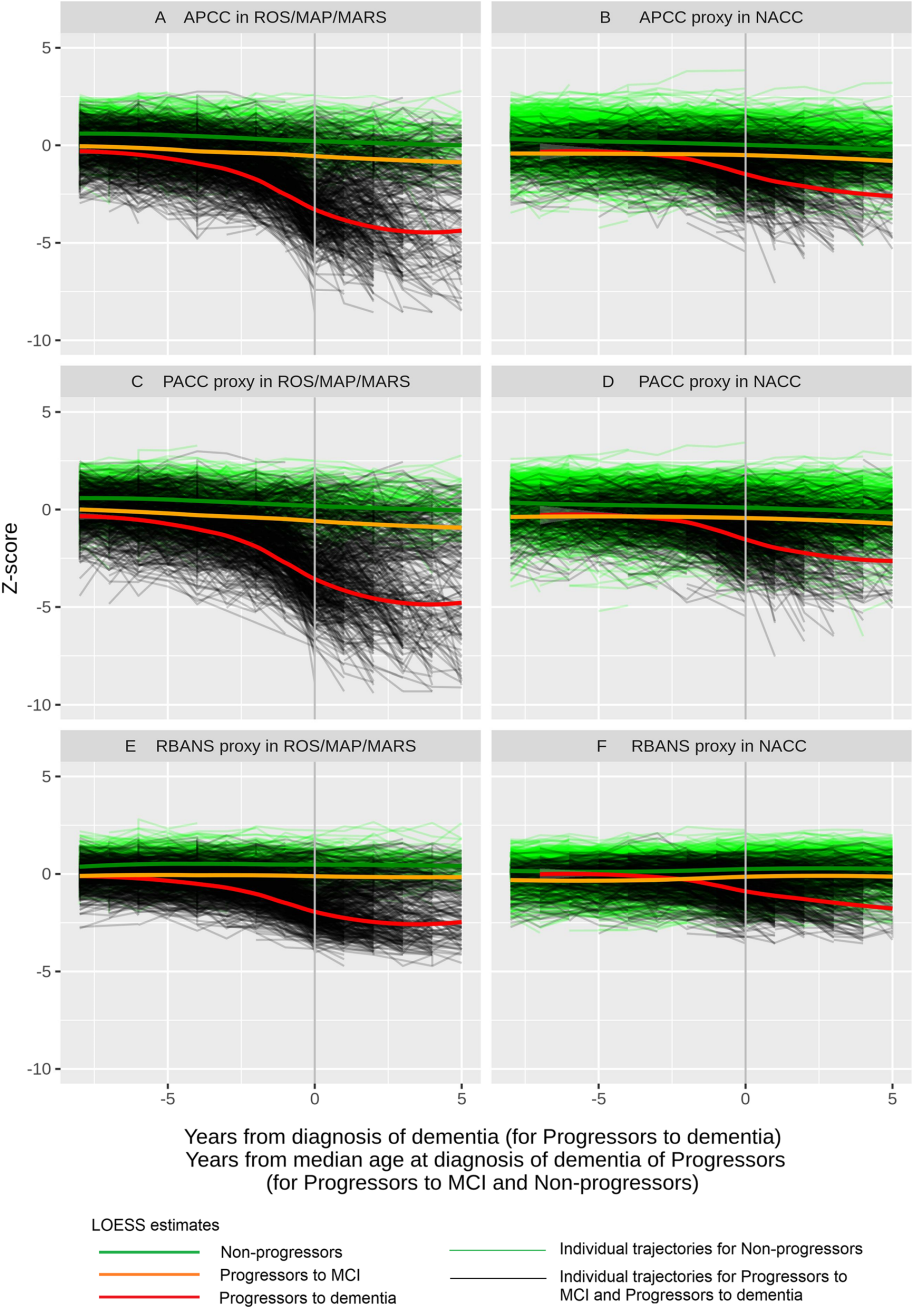
Cognitive composites over time by progressor status: Individual time profiles and LOESS estimates. APCC, Alzheimer’s Prevention Initiative Preclinical Composite Cognitive; LOESS, locally estimated scatterplot smoothing; MCI, mild cognitive impairment; NACC, National Alzheimer’s Coordinating Center; PACC, Preclinical Alzheimer Cognitive Composite; RBANS, Repeatable Battery for the Assessment of Neuropsychological Status; ROS/MAP/MARS, Religious Orders Study/Memory and Aging Project/Minority Aging Research Study. Trajectories are anchored at the time of diagnosis for progressors to dementia and aligned by the median age of progressors at the time of diagnosis of dementia for progressors to MCI and for non-progressors to MCI/dementia

Cognitive performance over time was investigated using the APCC in the ROS/MAP/MARS cohorts and the APCC proxy in NACC on the three categories of progressors to dementia due to AD, progressors to MCI (who did not progress to dementia during the time of observation), and non-progressors (to MCI, dementia, or both). The mean APCC (or its proxy) score at study entry was 64 in both ROS/MAP/MARS and NACC. Proxies for PACC and RBANS were also investigated. Observed patterns in cognitive decline, shown using standardized *z*-scores, were similar across the different cognitive composite measures (Fig. 1).

On average, progressors to only MCI and non-progressors showed a similar, linear, quite flat course in time, with the only difference between the two groups being that progressors to MCI (not to dementia) started at a lower cognitive level than non-progressors. By contrast, the average cognitive decline for progressors to dementia was not linear, starting at a low rate about 10 years before diagnosis and becoming steeper a few years before diagnosis, mainly during the MCI stage. The LOESS estimate for progressors to MCI (not to dementia) may have been impacted by a differential drop-out after the diagnosis of dementia due to AD.

Splitting by *APOE4* status in NACC (which contains most of the *APOE4* homozygote data) using the APCC, PACC, and RBANS proxies showed that the shape of the curve did not seem to depend much on the genotype if anchored at the time of diagnosis, with a steep decline in cognition occurring 2–4 years prior to the manifestation of dementia (Additional file 1: Fig. S1). Plotting the APCC data versus age showed that homozygotes started to decline cognitively at a younger age than heterozygotes and non-carriers (data not shown).

Taking all these results together, we concluded that the APCC decline was not linear and was mainly driven by how close the diagnosis of dementia was. When APCC was anchored at the time of diagnosis, the impact of genotype and age was minor. In addition, in earlier stages of the disease (i.e., more than 8 years before the diagnosis of dementia), late progressors behaved very similarly to non-progressors. We also confirmed that the PACC behaved similarly to the APCC.

### Modeling

#### Identification and evaluation of the TTE model

The data from ROS/MAP/MARS and NACC (total *N* = 9900, Table 2) that were used to fit the TTE model included a total of 2076 participants who progressed to MCI or dementia due to AD (*N* = 497 from ROS/MAP/MARS and *N* = 1579 from NACC, Table 2).

**Fig. 2:**
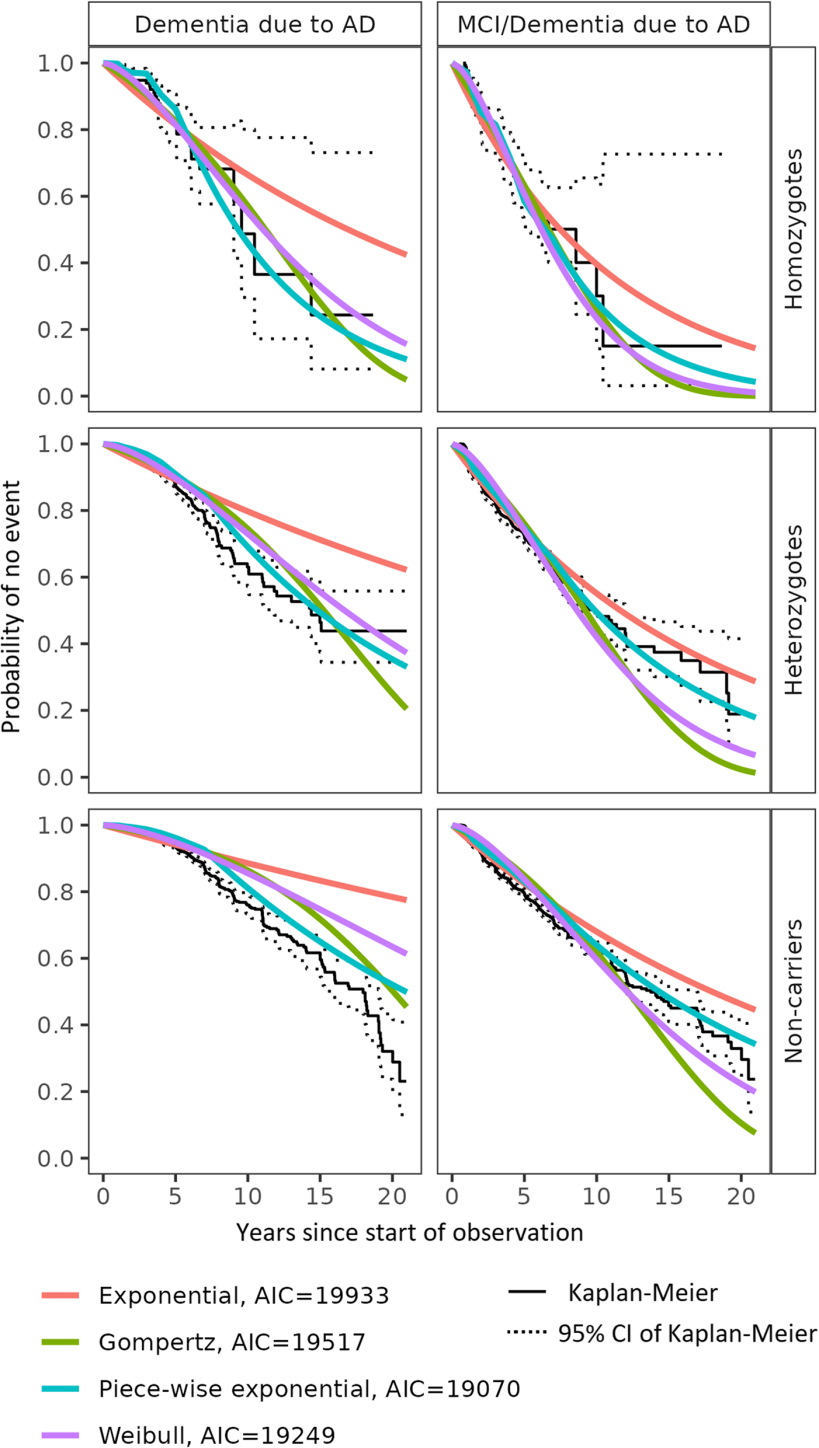
Probability of remaining cognitively unimpaired obtained by different survival functions for the TTE model. AD, Alzheimer’s disease; AIC, Akaike’s Information Criterion; CI, confidence interval; MCI, mild cognitive impairment; TTE, time to event. Kaplan-Meier curves: confidence limits are wide for homozygotes due to the small sample size. Non-genotyped subjects were assumed to be non-carriers

The model included factors for event type and genotype (Fig. 2). Having the event type as a factor allowed us to estimate both TTE endpoints, i.e., time to diagnosis of MCI or dementia due to AD (whichever is first) and time to dementia due to AD, with the same model.

The candidate models (Weibull, piece-wise exponential, exponential, and Gompertz) were investigated and compared visually by genotype and by using AIC. Figure 2 depicts the predictions of the probability to remain event-free (i.e., no diagnosis of MCI or dementia) from the four models overlaid with the KM estimates based on the observed data by genotype and shows that all models characterized well the observed data across genotypes. The models also included a submodel for the time to diagnosis of dementia due to AD (but not for the time to diagnosis of MCI). The AIC values for each of the models were 19,249 (Weibull), 19,070 (piece-wise exponential), 19,933 (exponential), and 19,517 (Gompertz), favoring the piece-wise exponential and Weibull models over the other two candidates. Since the AIC values were relatively close, we selected the Weibull model because of the higher flexibility and lower complexity of this model compared to the piece-wise exponential, which outweighed the slightly better performance of the latter.

Candidate individual factors that could explain the between-subject variability of the TTE were identified as APCC at baseline, years of education, *APOE* genotype, sex, and age at baseline. A backward elimination approach based on AIC was performed. The final model included interactions of event type with *APOE4* genotype, age at baseline, years of education, and APCC at baseline. Adding sex to the model did not improve the model fit.

#### Quality check of the Weibull model for the TTE

**Fig. 3:**
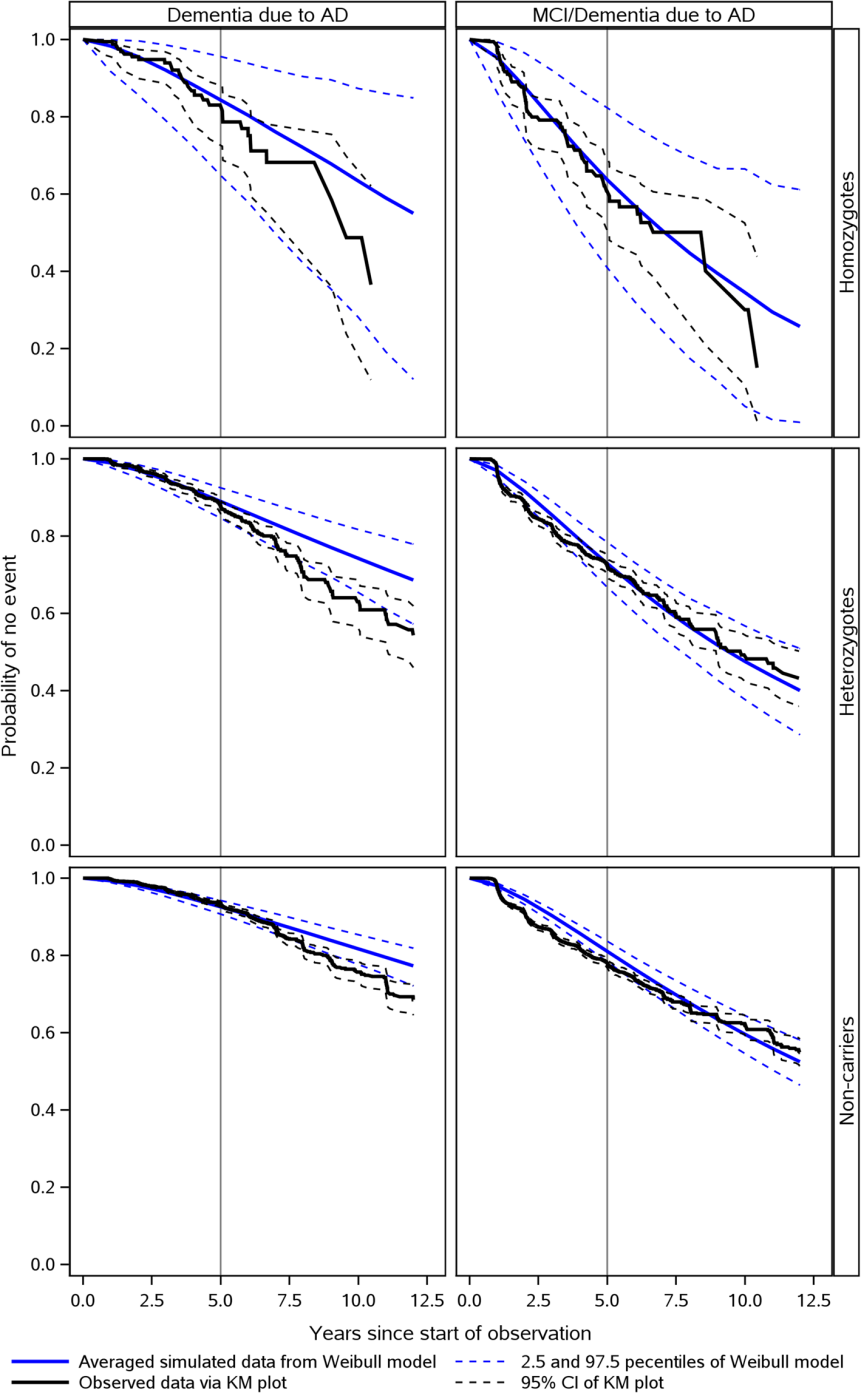
Model diagnostics of the TTE model (VPC). AD, Alzheimer’s disease; CI, confidence interval; KM, Kaplan-Meier; MCI, mild cognitive impairment; TTE, time to event; VPC, visual predictive check. Non-genotyped subjects were assumed to be non-carriers

A VPC comparing the observed and simulated data showed that the Weibull TTE model fit the data with good accuracy regardless of the type of event, i.e., diagnosis of MCI or dementia due to AD or diagnosis of dementia due to AD (Fig. 3). The adequate fit was confirmed by cross-validation (a model estimated on 50% of the data used to predict observations in the other 50%) (Additional file 1: Fig. S2). It should be noted, however, that the fit was less accurate for the diagnosis of dementia in the homozygote subpopulation, though this was not unexpected considering that it is much smaller than the other genotype subpopulations.

#### Identification and evaluation of the APCC models

The data from ROS/MAP/MARS used to fit the APCC models included 536 progressors and 1352 non/late progressors with available data on the APCC. Based on the data exploration, a power model was chosen to characterize the cognitive decline as measured by the APCC in the years before and after MCI or dementia diagnosis in the progressors subpopulation of the ROS/MAP/MARS cohorts.

A linear model was adequate to characterize the time course of the APCC score in the non/late progressors.

Candidate covariates tested for inclusion in the APCC models were APCC at baseline, *APOE4* status (homozygotes, heterozygotes, and non-carriers/non-genotyped), years of education, sex, age at baseline, and age at the time of the first MCI/AD diagnosis. The covariates selected for the final APCC models for progressors and non/late progressors are shown in Table 3.

**Table 3:** Covariates selected for the APCC models

	Progressors		Non/late progressors	
	Impacting baseline values^a^	Impacting the progression rate	Impacting baseline values^a^	Impacting the progression rate
APCC at baseline	X	X	X	X
*APOE4* status		X		X
Years of education	X	X	X	X
Age at baseline			X	X
Age at the time of event	X			

*APCC* Alzheimer’s Prevention Initiative Preclinical Composite Cognitive, *APOE4* apolipoprotein E ε4

^a^Covariates impacting the individual’s APCC value 12 years before the first diagnosis. Baseline covariates centered around observed medians: APCC logit-transformed and centered around the value 62, age around 74, and years of education around 16. *APOE4* status includes homozygotes, heterozygotes, and non-carriers/non-genotyped

**Fig. 4:**
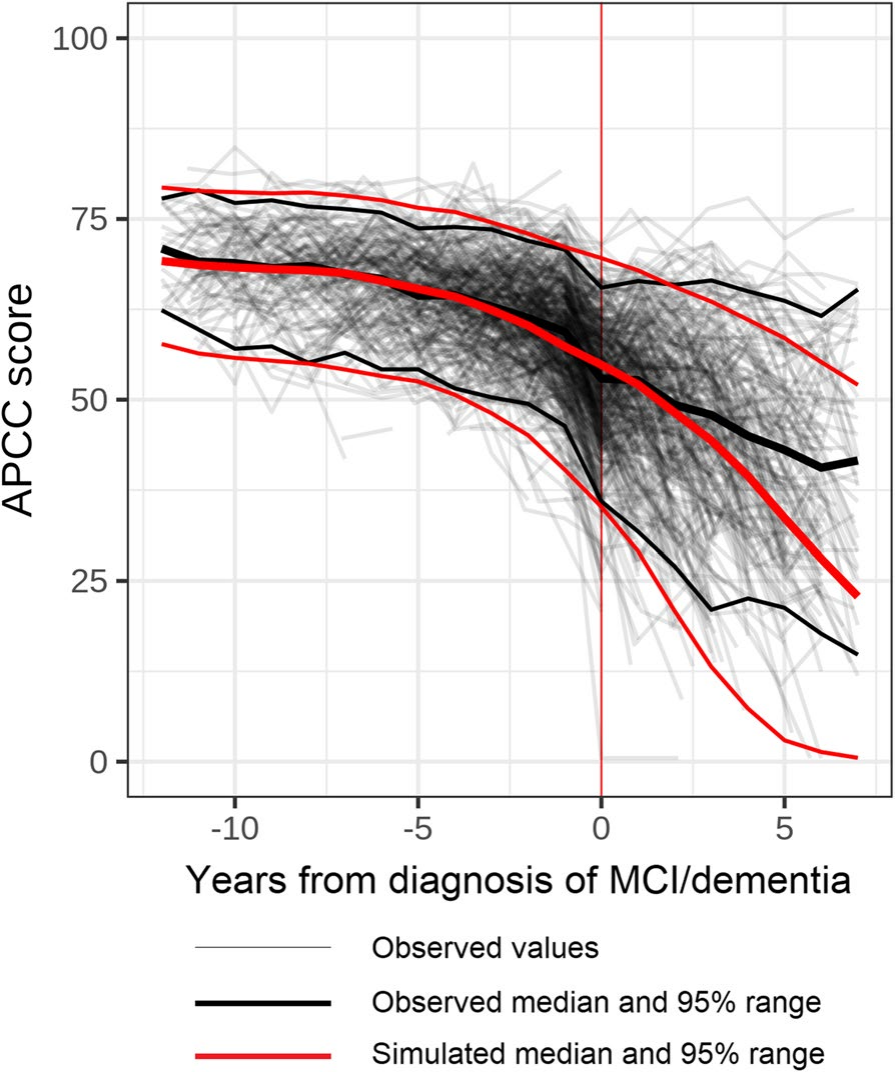
Model diagnostics of the APCC model for progressors to MCI/dementia due to AD (VPC). AD, Alzheimer’s disease; APCC, Alzheimer’s Prevention Initiative Preclinical Composite Cognitive; MCI, mild cognitive impairment; VPC, visual predictive check. Diagnosis of MCI/dementia: diagnosis of mild cognitive impairment or dementia due to AD

The adequacy of the models was assessed via VPCs based on 1000 replications. The simulated data reproduced the decline and variability of the APCC score reasonably well, indicating that the models adequately represent the data (Fig. 4 for progressors and Additional file 1: Fig. S3 for non/late progressors), especially in the time from 10 years before to 2 years since diagnosis. The assessment of the model fit beyond 2 years after diagnosis is difficult due to differential drop-out, and it is not within the scope of this study. In addition to the VPC, the NPDE indicated good adequacy of the models (data not shown).

**Fig. 5:**
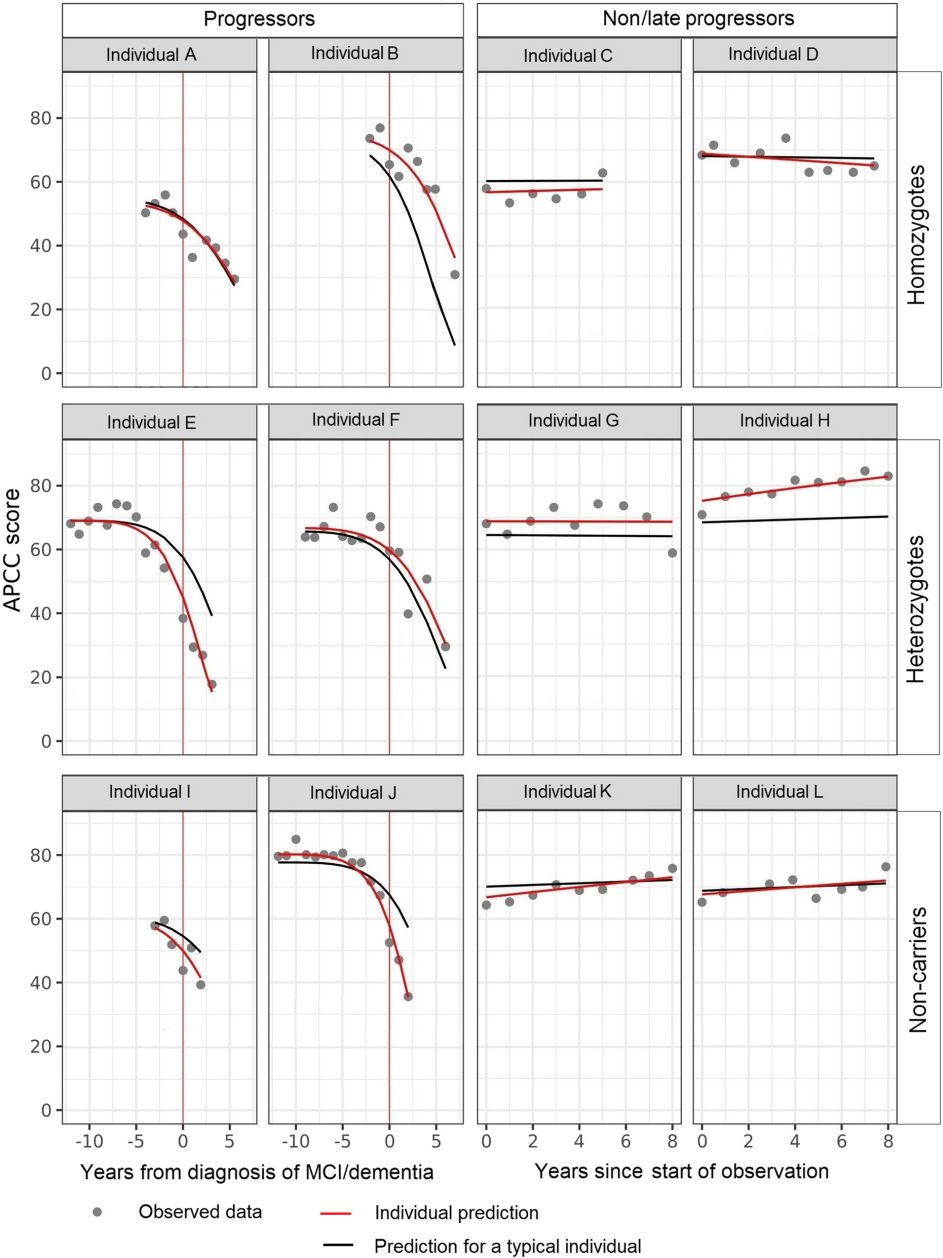
Examples of individual profiles of predictions and observed data. APCC, Alzheimer’s Prevention Initiative Preclinical Composite Cognitive; MCI, mild cognitive impairment. Both individual predictions and predictions for a “typical individual” take into account all model covariates; individual predictions are further adjusted by estimates of individual variability not explained by identified covariates

Figure 5 shows a comparison of typical and individual predictions and observed data (as well as NPDE for each data point) for a few randomly selected progressors and non/late progressors of different genotypes.

### Simulation platform

The simulation platform allowed us to generate a large set of virtual subjects defined by their demographic characteristics (see the “Methods” section) to explore the endpoints dynamics and dependencies. Clinical endpoints were simulated based on the TTE and APCC models described in the previous sections. Since the APCC score at baseline was simulated based on a model including age and years of education as factors, years of education was removed from the factors in the TTE model to avoid co-linearity with the baseline APCC score.

As part of the covariates, the distribution of baseline factors such as age in the trial population has a major impact on the event rates and the change in APCC over time. Firstly, we investigated the impact of the parameters of interest using the underlying simulated population based on bootstrapping (*n* = 100 repeats).

Our main objective was to understand the dependency/variation of the event risk at year 5 with respect to risk factors and to compare this with published results about *APOE4* homozygotes [19]. Another objective was to explore the factors/dynamics of the change in APCC and the resulting effect size in terms of the reduction of APCC decline compared to the control group.

To optimize the enrichment strategy of Generation Study 1, we examined the impact of restricting the age distribution on the power of the trial by investigating a 1:2:2 ratio of age groups 60–65, 65–70, and 70–75 years (mean age: 68.2 years) compared with the expected “natural” ratio of 3:2:1 (mean age: 65.4 years). Table 4 shows the results at the simulated population level (not from simulated clinical trials) for the two assumptions on the age distribution and the selected outcomes of interest. In the older population (1:2:2 ratio of age groups), the median TTE is shorter and the event risk rate at year 5 is higher (6.5 years and 40% risk for 1:2:2 vs 7.5 years and 34% risk for 3:2:1 for the control group specified by a HR of 1). In comparison, the estimated event risk rate at 5 years, as determined by the KM estimates for homozygotes, was 38% based on pooled data from ROS/MAP/MARS, NACC, and ADNI (mean age: 67.8 years). Thus, if a clinical trial population includes a smaller proportion of younger individuals (no more than 20% in the age range of 60–65 years), the event rate may be higher than expected.

**Table 4:** Endpoint characteristics for two different age distributions within the selected age range

HR	Age distribution 3:2:1			Age distribution 1:2:2		
	Median TTE, years	Event risk at year 5	Effect size of APCC change from BL to year 5	Median TTE, years	Event risk at year 5	Effect size of APCC change from BL to year 5
0.60	10.705	0.228	0.2209	9.095	0.269	0.2319
0.65	10.045	0.243	0.1916	8.830	0.286	0.2008
0.67	10.000	0.248	0.1827	8.500	0.293	0.1860
0.70	9.640	0.258	0.1752	8.500	0.304	0.1765
0.75	9.230	0.273	0.1364	8.000	0.321	0.1325
1.00	7.500	0.343	0.0000	6.500	0.399	0.0000

*APCC* Alzheimer’s Prevention Initiative Preclinical Composite Cognitive, *BL* baseline, *HR* hazard ratio, *TTE* time to event

The derived effect sizes in terms of reduction of the change in APCC score from baseline to year 5 were low, ranging from 0.23 for a HR of 0.60 (40% risk reduction) to 0.13 for a HR of 0.75 (25% risk reduction), even for the older population (1:2:2, Table 4).

### Clinical trial simulation

We implemented a clinical trial simulation platform to sample participants of clinical trials from the simulated population under various options. The platform allows the following:

- Sampling of participants of clinical trials controlling for the age distribution within the age range of 60–75 years
- Varying the recruitment pattern and duration
- Varying drop-out patterns and probabilities
- Selecting the total sample size, the ratio between active and control groups, and the follow-up time
- Calculating power to demonstrate a treatment effect in at least one endpoint based on different analysis methods for the two endpoints (TTE and APCC)

The results for a family-wise type 1 error rate of 5% and different scenarios to distribute the type 1 error (using a simple Bonferroni adjustment for testing two hypotheses for the two endpoints) are summarized for the age distribution of 1:2:2 in Table 5.

**Table 5:** Power of clinical trial replicates (1000 simulation runs) for the 1:2:2 age distribution

HR	Power given the distribution (%) of type 1 error rate for TTE/APCC				
	100%/0% single primary endpoint: TTE	80%/20%	50%/50%	20%/80%	0%/100% single primary endpoint: APCC
0.60	0.959	0.952	0.943	0.914	0.798
0.65	0.877	0.855	0.822	0.777	0.653
0.67	0.840	0.819	0.785	0.735	0.581
0.70	0.752	0.723	0.693	0.645	0.525
0.75	0.581	0.556	0.499	0.439	0.337

*APCC* Alzheimer’s Prevention Initiative Preclinical Composite Cognitive, *HR* hazard ratio, *TTE* time to event

The power of the TTE endpoint alone was above 75% for a HR of 0.70 to 0.60 (two-sided log-rank test). The overall power was only slightly lower for the 80%/20% distribution of the alpha between TTE and APCC. The power for the APCC endpoint alone (two-sided *t*-test) was consistently lower compared to the power for the TTE endpoint alone. Also, the overall power was lower for the 20%/80% distribution of the family-wise type 1 error rate between TTE and APCC compared to the 80%/20% distribution.

Based on the simulation results, we incorporated the 1:2:2 age distribution in the design of Generation Study 1, thereby restricting the recruitment of the lowest age group (60–65 years) to 20% of the target sample size. For the primary statistical analysis, an initial distribution of the family-wise type 1 error rate was set to 20% for testing the primary hypothesis on the APCC and to 80% for the TTE within a graphical procedure. This approach comprised the dual endpoints as well as the key secondary endpoint of Generation Study 1 (Clinical Dementia Rating Scale - Sum of Boxes [CDR-SOB]) and was adjusted for testing multiple endpoints and to allow alpha propagation after the rejection of the null hypothesis for one endpoint to another. In addition, a variable follow-up time of 5 to 8 years was planned to further increase the power of the TTE endpoint.

Additional simulations tailored to the final design were implemented to assess the power under realistic assumptions regarding recruitment and drop-out patterns (data not shown). The time to drop-out was assumed to be exponentially distributed and the drop-out rate to be 30% in 5 years based on experience with clinical trials in early AD and subject matter expert input on the target population. The overall power at the projected analysis time was acceptable (ranging from 75 to 96%) for HRs of 0.70 to 0.60, which corresponds to a risk reduction of 30 to 40%.

## Discussion

Using longitudinal model-based analyses of historical cohort data, we have developed a simulation platform to inform the design of preclinical AD studies, enrolling cognitively unimpaired individuals with a potentially elevated risk of developing AD in the coming years. This quantitative approach facilitated the investigation of a dual endpoints strategy capturing both subtle continuous cognitive decline and discrete clinically relevant events in this population. Our simulations allowed us to optimize the performance of the dual endpoints, for example, leading to a limitation of no more than 20% of participants in the youngest age group of 60 to 65 years [8].

Our main conclusion is that a clinical trial in a cognitively unimpaired population enriched by the *APOE* genotype and older individuals needs to be large and have a long observation period of at least 5 years to be able to detect clinically relevant treatment effects. In simulations, the high proportion of non/late progressors in this population led to only small average changes in the APCC over time, accompanied by a high variability in the placebo group, translating to small effect sizes (even for large treatment effects, e.g., a HR of 0.60, representing a 40% risk reduction). The other explored composites PACC and RBANS performed similarly to the APCC score in terms of monitoring the progression of AD (exploratory analyses). These results suggest that none of the three investigated cognitive composite measures (1) seems to be sensitive enough to capture the slowing of progression due to treatment effects in cognitively unimpaired individuals and (2) will reduce the required sample size or duration when used as the stand-alone primary endpoint in clinical trials targeting the preclinical phase of the disease compared to a TTE endpoint. This is in line with Cummings (2019) who, interpreting the results from Donohue et al. (2017), concluded that even in FDA stage 2 of preclinical AD, very large sample sizes observed for extended periods of time would be needed to demonstrate a drug-placebo difference [21–23].

The proposed dual endpoints approach allows the examination of drug effects on two separate measures of disease progression: TTE and cognitive decline (APCC). We included a TTE endpoint because postponing the onset of MCI and/or dementia due to AD represents an important and meaningful clinical outcome with high face validity which meets regulatory requirements. In a clinical trial setting, the definition of an event can be standardized (as it was for both generation studies) by using an adjudication committee. Furthermore, the TTE endpoint not only captures information about the incidence of events per se, but it also captures the time required for the event to occur as a continuous outcome variable. Finally, the TTE endpoint reached reasonable power, that is, at least 80%, in various simulated scenarios. In an event-driven design, the variable treatment duration allows an increase in power (since the time at risk is increased in participants who have been randomized early) without delaying the availability of final study readouts and can also facilitate and improve confidence in interim analyses.

In addition to the TTE endpoint, using a composite cognitive endpoint is expected to capture subtle changes in cognition even before the diagnosis of MCI in individuals who subsequently progress to the clinical stages of AD, thus supporting the dual endpoints approach. The APCC score was developed as a combination of well-established, validated neuropsychological test scores to evaluate treatments in prevention settings [9]. As such, it is likely to be appropriate for the evaluation of preclinical AD treatments in prevention trials. In contrast to the PACC and other cognitive composites currently used in preclinical AD [16], the APCC was developed using an empirical strategy supported by a theoretical understanding of preclinical cognitive changes. There is some evidence that such an empirically based composite is highly sensitive to these changes over time [9].

Although the APCC score showed high variability when calculated retrospectively from the available cohort data, this cognitive measure may be less variable in a clinical trial setting where the population is more homogeneous. Ensuring standardized administration and scoring rules across centers and countries will also help decrease the variability. In any case, it should be noted that our results were consistent across the three composite cognitive scales that were considered (APCC, PACC, and RBANS) as well as across cohorts (ROS/MAP/MARS and NACC), strongly suggesting that the findings on the APCC from the ROS/MAP/MARS cohorts may hold in a more general framework.

A trial using the dual endpoints approach requires a positive result in at least one endpoint to be successful. In this study, there was a slight loss in power of the dual endpoints approach compared to TTE as a stand-alone endpoint (Table 5). However, the fact remains that a dual endpoints approach is preferable as it allows for the examination of drug effects on two clinically relevant disease progression measures and mitigates the risk of an uninformative clinical trial if one of the two primary outcomes does not perform as expected. In addition, the loss of power was minimal even with a simple Bonferroni adjustment of the family-wise type 1 error rate.

### Limitations

Although different historical data sources have been included in this study, the question about the external validity of these data remains unanswered, i.e., how representative the data are of the wider population. In addition, the number of homozygotes in the historical data was small. This limitation in terms of genotype was bypassed by the graphical exploration of longitudinal data: anchoring the APCC trajectories at the time of the diagnosis of dementia revealed that the time course of cognitive decline in AD progressors was similar across different risk groups as determined by their genotype. This is in line with results from Bonham (2016) reporting that the *APOE4* genotype influences the risk of progression to MCI and dementia due to AD as a function of age [19]. As a consequence, data from all progressors (regardless of genotype) was used to inform both models.

Although NACC and ROS/MAP/MARS included a large number of participants with a long follow-up and a rich variety of cognitive measures, there were no biomarker data available to inform the modeling. Biomarker assessments, including amyloid status, are available from ADNI, but longitudinal data in this cohort are limited, especially in the target population of *APOE4* homozygotes. Because our models could not assess the predictive value of amyloid status or other biomarkers (e.g., biomarkers of tauopathy or neurodegeneration), our approach may not be appropriate under every circumstance (e.g., in a clinical trial design including biomarker assessments and/or using biomarkers to enrich the study population). This was the case for Generation Study 2 for which a simulation was not possible since the inclusion criteria required the *APOE4* heterozygote participants to have elevated amyloid. Our approach may reach different conclusions and allow for a smaller sample size with such designs, as the power to detect a treatment effect based on composite cognitive endpoints used as single primary endpoints is potentially higher in a more advanced population enriched by biomarkers [16].

In addition, the conceptual setup of the simulation implicitly favored the TTE over the APCC. The assumed relationship between the endpoints did not allow us to directly control the treatment effect on the APCC time course, as it was driven by the HR selected for the TTE. The association between the TTE and the APCC over time has been simplified, since it was captured using a dichotomized TTE (early vs non/late progressors) as opposed to a more complex functional relationship reflecting a possible correlation between the endpoints. Also, the TTE model was developed assuming that the drop-out occurs at random, which might not be correct. Nevertheless, quality checks of the selected endpoint models showed a very good fit to the observed data. We confirmed the structures of the APCC models by simulation-based approaches (VPC and NPDE) and that of the TTE models by additional cross-validation.

The same data (ROS/MAP/MARS) has been used to develop the novel endpoint APCC (based on a data-driven procedure) and to fit a longitudinal model for the APCC. As previously mentioned, we observed similar patterns for two other proposed composites in the ROS/MAP/MARS cohorts and for all three investigated composites in NACC. However, it should be noted that we have not developed models for the PACC and RBANS. Comparisons across composites were constructed using proxies and were only based on observed data using visual inspection and simple MSDR analyses.

Finally, although the simulation platform is based on models with good internal/external validity, their performance on new data has not been assessed.

### Dual endpoints approach compared with other proposals

Several cognitive composite endpoints based on existing cognitive scales have been proposed based on the assumption that including a measure that is sensitive to early changes will increase the power of preclinical AD studies. We focused on the APCC, which has been developed for this purpose, but data exploration and trial simulations did not confirm this claim. The effect sizes were low, and the resulting power was not sufficient for a stand-alone primary endpoint. This contrasts with results reported for the PACC by Donohue et al. and Li et al. based on data from ADNI [23, 24].

Analyses by Donohue et al. found an effect size for the modified PACC of approximately 0.5 at year 4 for amyloid-positive versus amyloid-negative cognitively unimpaired individuals, which could be seen as a benchmark for the largest possible effect size of a potential treatment. In our simulations for *APOE4* homozygotes (not enriched by amyloid status), the effect sizes at year 5 comparing active treatment versus placebo were much smaller even in the most optimistic scenarios.

Like Donohue et al., Li et al. suggested that a smaller study (2-year duration) could reach reasonable power. In addition, they specifically assessed the relative efficiency of the TTE and continuous measures of cognition (PACC) in pre-symptomatic AD [24]. According to their results, the power was approximately doubled with models of repeated cognitive assessments (mixed models of repeated measures) compared with the time-to-progression analysis (Cox proportional hazards model, time to MCI diagnosis). This contrasts with the power we obtained for the APCC based on our clinical trial simulations, which was consistently lower than the TTE. Moreover, the power results for the TTE reported by Li et al. are much lower than those obtained with a standard sample size calculation tool (PASS version 11, 2012). Using a total sample size of 1000 with a 1:1 randomization ratio, the type 1 error rate of 5%, a drop-out pattern as described in Li et al.’s publication, and a progression rate to MCI in the placebo group of 24% in 8 years led to a power of 22%, 46%, and 73% for treatment effects of 20%, 30%, and 40%, respectively; the corresponding power in Li et al. was 19%, 32%, and 50% (Table 4 [24]).

Our approach differs from that of Donohue et al. and Li et al. in a number of aspects. We used a much larger data source, with more than 10,000 individuals for the TTE model (vs 445 in Donohue et al. and 163 in Li et al.). Furthermore, both Donohue et al. and Li et al. examined a biomarker-enriched population (i.e., with elevated amyloid) whereas we examined a broader population enriched by age and *APOE* genotype, which is less advanced and may be more variable. A more advanced amyloid-positive population may decline much faster compared to *APOE4* homozygotes in the age range of 60–75, and cognitive composites may therefore perform better. Finally, the PACC could be more sensitive and have lower variability than the APCC, although in our study, the two composites showed a similar pattern of progression. Of note, the good performance of the PACC in ADNI was not reproduced in ROS/MAP/MARS or in NACC, in line with what has been observed by others [22].

An alternative hypothesis is that the conceptual differences in the modeling and simulation approach may have driven the differences in outcomes. As noted in the limitations, our approach implicitly favors the TTE endpoint (since the outcome of TTE informs the selection of the APCC model) whereas the approach of Li et al. implicitly favors the continuous cognitive endpoint (diagnosis is derived from the outcomes of the continuous cognitive measures). Other details of the modeling are also different, which may add to although are not likely to drive the discrepancies. We used a parametric function to define the TTE endpoint, which is a more informative approach than the semi-parametric Cox model (that only uses the information at the time of the event) and which, contrary to a Cox regression, can use any functional form to model a covariate effect.

The findings of Insel et al. are more in line with ours in terms of the required sample size and study duration [22]. In their study, the PACC was used to investigate the development of MCI in 1120 individuals who were cognitively unimpaired and amyloid positive at study entry (with the exclusion of participants with high cognitive performance). In 4-year trials, assuming a 25% slowing of cognitive decline in the treatment group, the required sample size to reach 80% power was 2000 per group combining all cohorts. Combining a longer duration together with a higher drug effect (6-year trials and 35% treatment effect), the required sample size decreased to 300 per group on average.

The lack of sensitivity and limitations of existing composite cognitive scales in the early stages of AD have also been put forward by Schneider and Goldberg [25]. These authors propose novel cognitive measures that may be more reliable and sensitive to very subtle changes, and thereby yield evidence of impairment and progression unseen in the analyzed historical cohorts; these, however, still need to be validated [26, 27].

## Conclusions

As long as there is no evidence from prospectively collected data that a composite cognitive measure is sufficiently sensitive in detecting and tracking early changes in cognitive performance to be used as a single primary endpoint in clinical studies of preclinical sporadic AD, we recommend the dual endpoints approach. This includes a TTE endpoint along with a composite cognitive endpoint, together with an adequate adjustment for testing hypotheses on multiple endpoints. In line with the results from several studies [21, 22] but contrasting with reports from other researchers [24], we conclude that clinical trials in preclinical AD need to have a large population and a long duration. Further research with a similar approach may determine whether and to what extent enrichment with biomarkers or more sensitive and reliable cognitive tests could reduce the sample size or duration requirement. Trial simulation based on endpoint models is a powerful tool to optimize clinical trial designs, leading to a more realistic and scientifically driven strategy and, potentially, a greater probability of success.

## Supplementary Information

**Additional file 1: **Supplemental material. **Fig. S1.** Cognitive composites over time by genotype: individual time profiles and LOESS estimates for progressors to dementia. **Fig. S2.** Cross validation of TTE model structure. **Fig. S3.** Model diagnostic of the APCC model for non-progressors (VPC).

## Data Availability

The data that support the findings of this study are from historical cohorts, including the Rush Alzheimer’s Disease Center (RADC) (ROS/MAP/MARS data can be requested at https://www.radc.rush.edu), the Alzheimer’s Disease Research Centers of the National Alzheimer’s Coordinating Center (NACC) (https://www.alz.washington.edu), and the Alzheimer’s Disease Neuroimaging Initiative (ADNI) (http://adni.loni.usc.edu); however, restrictions apply to the availability of these data, which were used under license for the current study, and so are not publicly available. Requests for data should be directed to the respective centers.

## Abbreviations

AD: Alzheimer’s disease
ADNI: Alzheimer’s Disease Neuroimaging Initiative
AIC: Akaike’s Information Criterion
APCC: Alzheimer’s Prevention Initiative Preclinical Composite Cognitive
API: Alzheimer’s Prevention Initiative
APOE: Apolipoprotein E
CDR-SOB: Clinical Dementia Rating Scale - Sum of Boxes
EMA: European Medicines Agency
FDA: Food and Drug Administration
HR: Hazard ratio
KM: Kaplan-Meier
LOESS: Locally estimated scatterplot smoothing
MAP: Memory and Aging Project
MARS: Minority Aging Research Study
MCI: Mild cognitive impairment
MSDR: Mean-to-standard deviation ratio
NACC: National Alzheimer’s Coordinating Center
NPDE: Normalized prediction distribution errors
PACC: Preclinical Alzheimer’s Cognitive Composite
RADC: Rush Alzheimer’s Disease Center
RBANS: Repeatable Battery for the Assessment of Neuropsychological Status
ROS: Religious Orders Study
TTE: Time to event
VPC: Visual predictive check
