# Supplementary material for: Rationale for the selection of dual primary endpoints in prevention studies of cognitively unimpaired individuals at genetic risk for developing symptoms of Alzheimer’s disease
Source: Alzheimers Res Ther. 2023 Mar 6;15:45. doi: 10.1186/s13195-023-01183-z (PMC9987044; doi:10.1186/s13195-023-01183-z)
Supplement: Supplementary file 1 — Additional file 1: Supplemental material. Fig. S1. Cognitive composites over time by genotype: individual time profiles and LOESS estimates for progressors to dementia. Fig. S2. Cross validation of TTE model structure. Fig. S3. Model diagnostic of the APCC model for non-progressors (VPC). [file 13195_2023_1183_MOESM1_ESM.docx]

**Supplemental Material**

**Proposed title:** **Rationale for the selection of dual primary endpoints in prevention studies of cognitively unimpaired individuals at genetic risk for developing symptoms of Alzheimer’s disease**

**Authors:** Angelika Caputo, Amy Racine, Ines Paule, Pierre N. Tariot, Jessica B. Langbaum, Neva Coello, Marie-Emmanuelle Riviere, J. Michael Ryan, Cristina Lopez Lopez, and Ana Graf

Further details on the Alzheimer’s Prevention Initiative Preclinical Composite Cognitive (APCC) longitudinal models

Both APCC models were fit with NONMEM version 7.3.0

1. The APCC model for progressors is mathematically described as follows:

$logit\left( {APCC}_{it} \right)={logitBSL}_{i}-\frac{{rate}_{i}}{1000}\cdot time^{\theta_{3}}+\epsilon_{it}$

${logitBSL}_{i}=\theta_{1}+\theta_{4}\cdot\left( logit\left( \frac{{APCC0}_{i}}{100} \right)-logit\left( \frac{62}{100} \right) \right)+\eta_{1i}$

${rate}_{i}=\theta_{2}\cdot\theta_{5}^{{APOE}_{i}}\cdot\left( \frac{{Educ}_{i}}{16} \right)^{\theta_{6}}\cdot\left( \frac{{APCC0}_{i}}{62} \right)^{\theta_{7}}\cdot e^{\eta_{2i}}$

$time$ is defined as the number of years, counting from 12 years before the first event (mild cognitive impairment [MCI] or dementia);

*i* denotes the individual and *t* indexes the time of observation;

$APCCO$is the observed APCC score at baseline;

$APOE$is an indicator of being an apolipoprotein E ε4 (*APOE4)* carrier (1 for carriers, 0 for non-carriers);

$Educ$ is the duration of education (in years);

*rate i* is the rate of decline of an individual;

$\theta_{1}$ is an estimate of the APCC score 12 years before the first diagnosis (on a logit scale) of a typical progressor, with the observed APCC at baseline equal to 62 (median of progressors in the Rush cohorts), estimated as 0.581;

$\theta_{2}$ is the rate of decline in APCC of a typical progressor, a non-carrier of *APOE4*, with 16 years of education and a baseline APCC equal to 62, estimated as 0.0148;

$\theta_{3}$ is the exponent for the effect of time, fixed to 4 (because of a lack of information in the data);

$\theta_{4}$ is the covariate effect of baseline APCC on the APCC 12 years prior to the first event, estimated as 0.814;

$\theta_{5}$ is the multiplier for the rate of decline in the *APOE4* carriers, estimated as 1.11;

$\theta_{6}$ is the covariate effect of the years of education on the rate of decline, estimated as −0.918;

$\theta_{7}$ is the covariate effect of baseline APCC on the rate of decline, estimated as 2.62;

$\eta_{1}$ and $\eta_{2}$ are independently normally distributed subject-specific random effects for APCC 12 years prior to the first event and for the rate of APCC change, respectively, with zero mean and standard deviations, estimated as 0.117 and 0.693;

$\epsilon$ is a normally distributed random effect of the residual variability, with zero mean and standard deviation, estimated as 0.227.

1. The APCC model for non-progressors is mathematically described as follows:

$logit\left( {APCC}_{it} \right)={logitBSL}_{i}+\frac{{rate}_{i}}{1000}\cdot time+\epsilon_{it}$

${logitBSL}_{i}=\theta_{11}+\theta_{13}\cdot\left( Educ-16 \right)+\theta_{14}\cdot\left( Age-74 \right)+\eta_{3i}$

$${rate}_{i}=\theta_{12}+\theta_{15}\cdot APOE+\theta_{16}\cdot\left( Age-74 \right)+\eta_{4i}$$

$time$ is defined as the number of years, counting from the start of the observation;

$ApoE$is an indicator of being an *APOE4* carrier (1 for carriers, 0 for non-carriers);

$Educ$ is the duration of education (in years);

$Age$ is the age at baseline;

$\theta_{11}$ is an estimate of the APCC score at baseline (on a logit scale) of a typical non-progressor, with 16 years of education and an age of 74 years at baseline (median of progressors in the Rush cohorts), estimated as 0.69;

$\theta_{12}$ is the rate of change in APCC of a typical non-progressor, a non-carrier of *APOE4*, and an age of 74 years at baseline, estimated as 10.4;

$\theta_{13}$ is the covariate effect of education on baseline APCC, estimated as 0.0355;

$\theta_{14}$ is the covariate effect of baseline age on baseline APCC, estimated as −0.0097;

$\theta_{15}$ is the difference in the rate of APCC change in the *APOE4* carriers, estimated as −10.2;

$\theta_{16}$ is the covariate effect of baseline age on the rate of APCC change, estimated as −1.17;

$\eta_{3}$ and $\eta_{4}$ are independently normally distributed subject-specific random effects for baseline APCC and for the rate of APCC change, respectively, with 0 mean and standard deviations, estimated as 0.221 and 23.8;

$\epsilon$ is a normally distributed random effect of the residual variability, with zero mean and standard deviation, estimated as 0.163.

Further details on the time to event (TTE) model

1. Four candidate models were investigated and compared visually by genotype and using Akaike’s information criterion (AIC). These were Weibull, piece-wise exponential, exponential and Gompertz. For the piece-wise model, eight pieces were considered on a time interval of 20 years.
2. The TTE model selected was a Weibull model, which is mathematically described as follows:

$f\left( x \right)=\frac{k}{\lambda'} \left( \frac{x}{\lambda'} \right)^{k-1}e^{-\left( \frac{x}{\lambda'} \right)^{k}}$ where:

- $\lambda={(\lambda^{'})}^{-k},$
- $\lambda'=exp(\mu)=\exp\left( X\beta\right)$, where $\beta$represents the covariates, which are:
  - *Genotype (1 for carrier or 0 for non-carrier)*
  - *Type of event (2 for Alzheimer’s disease [AD] or 1 for MCI or dementia due to AD)*
  - *Type of event * log(Baseline age/60)*
  - *Type of event * log(Years of education)*
  - *Type of event * log(Baseline APCC/60)*
- $k=1/\sigma$, where $\sigma$ is a scale parameter (estimated by the statistical software)

*x* denotes the time to event;

*μ* is the average time to event or time to event of the population;

*λ* is a scale parameter defined as a function of the covariates and representing how spread is the Weibull distribution.

The model was fit with the PROC LIFEREG procedure in SAS using Weibull as the link function.

The intercept was estimated to be 5.4238.

The effect of the *Type of event* was estimated to be −1.0229 for MCI or dementia due to AD with respect to dementia due to AD.

The effect of the *genotype* was estimated to be 0.3409 for carriers with respect to non-carriers.

The effect of a change in units in years of baseline age (log scale) was −2.8847.

The effect of a change in units in years of education (log scale) was −0.6390.

The effect of a change in units of baseline APCC (log scale) was 3.2901.

The scale parameter was set to 0.6774 and the Weibull shape to 1.4762 (these two parameters are fixed by the SAS procedure).

Supplemental Figures

**Supplemental Figure S1:** Cognitive composites over time by genotype: individual time profiles and LOESS estimates for progressors to dementia
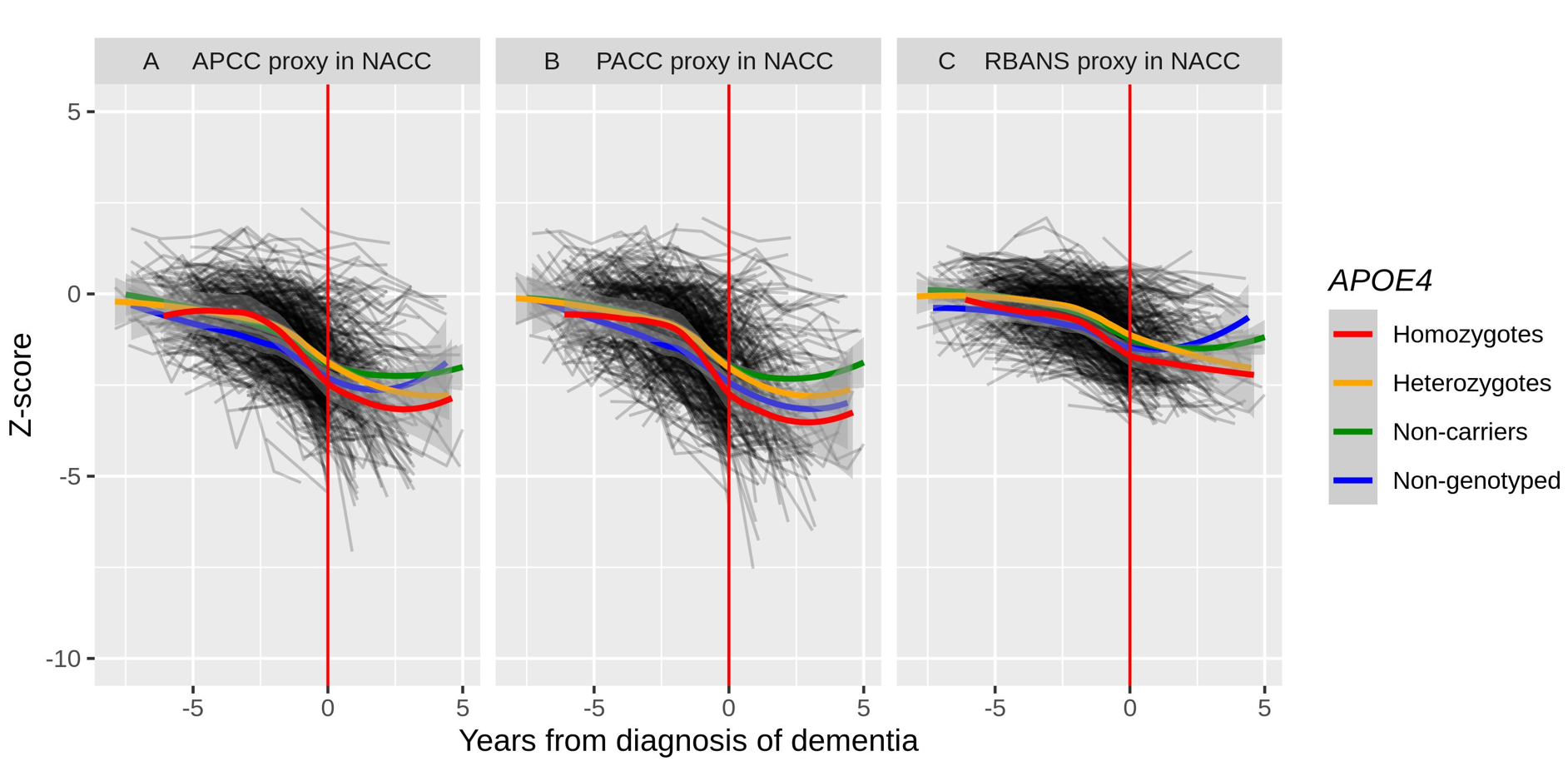


| LOESS, locally estimated scatterplot smoothing; NACC, National Alzheimer’s Coordinating Center; APCC, API preclinical cognitive; PACC, Preclinical Alzheimer Cognitive Composite; RBANS; Repeatable Battery for the Assessment of Neuropsychological Status; trajectories are anchored at the time of diagnosis of dementia |
| --- |

**Supplemental Figure S2:** Cross validation of TTE model structure


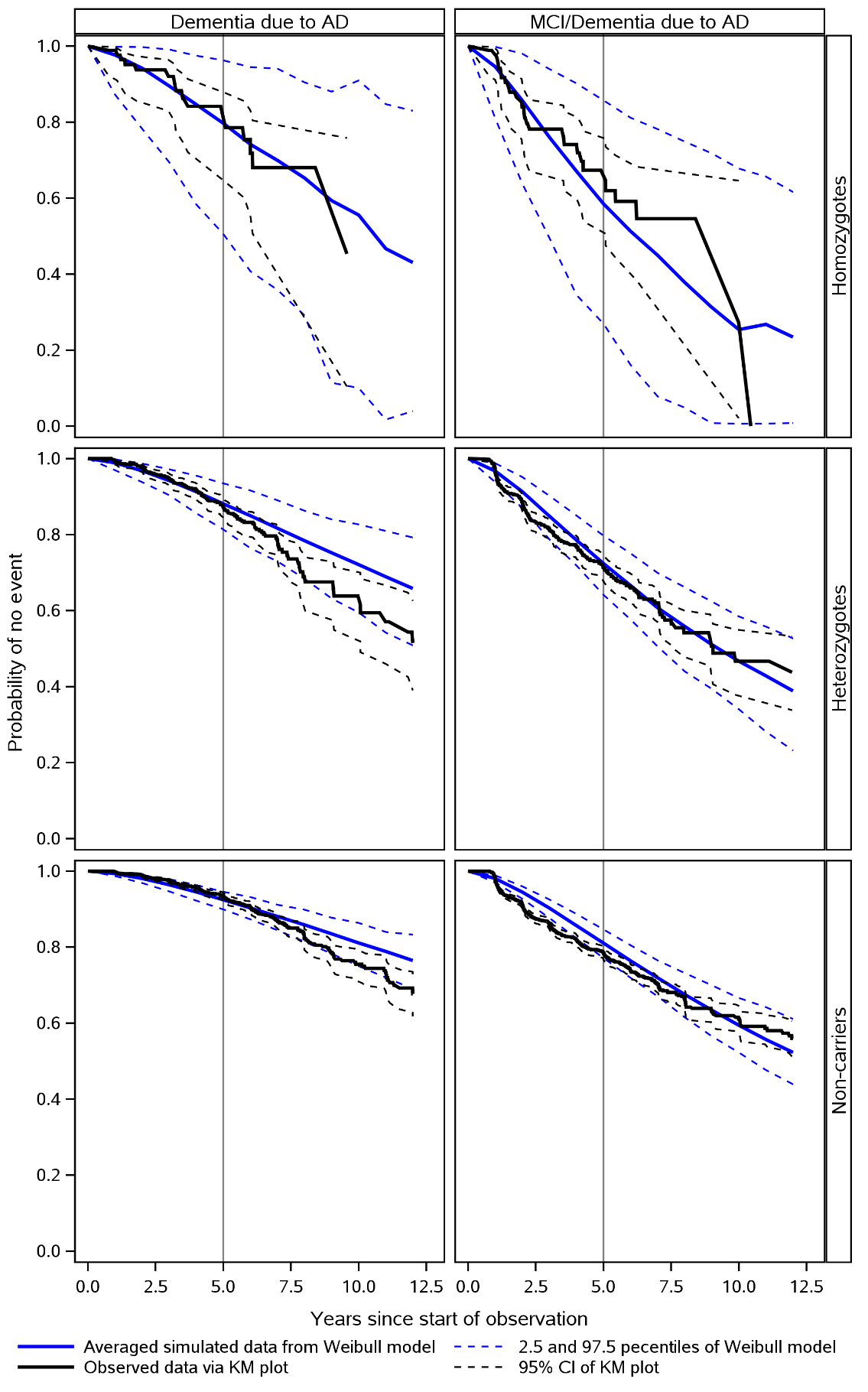


| AD, Alzheimer’s Disease; CI, confidence interval; MCI, mild cognitive impairment; TTE, Time to event. Non genotyped subjects are assumed to be non-carriers. Model built based on 50% of the data; prediction made for the other 50%. |
| --- |

**Supplemental Figure S3:** Model diagnostic of the APCC model for non-progressors (VPC)


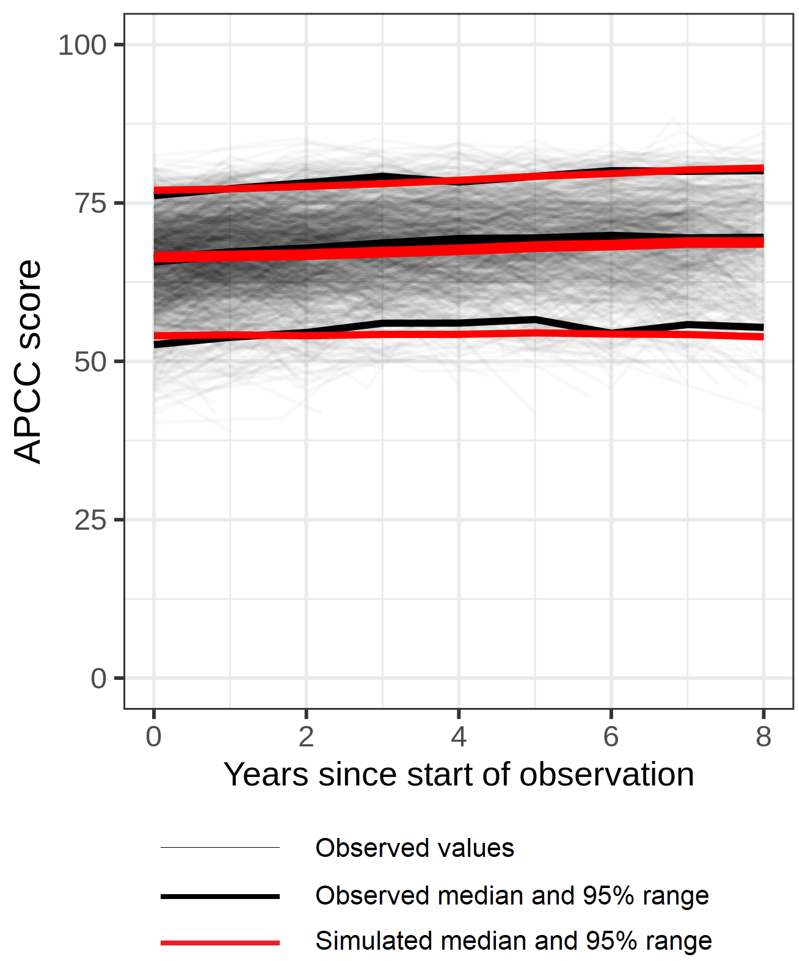


APCC, Alzheimer’s Prevention Initiative Composite Cognitive; VPC, Visual Predictive Check.
